# Optimization of load-bearing parameters for anisotropic nodes and prefabricated wall panels in prefabricated shear wall structures

**DOI:** 10.1371/journal.pone.0318521

**Published:** 2025-03-17

**Authors:** Jun Zhao, Libo Wang, Tengye Ma

**Affiliations:** School of Civil and Architectural Engineering, Anyang Institute of Technology, Anyang, China; University of Technology Sydney, AUSTRALIA

## Abstract

As the global construction industry develops, prefabricated buildings are gradually emerging and widely used. However, the bearing capacity of anisotropic nodes and prefabricated wall panels in prefabricated shear wall structures remains a technical challenge that restricts their widespread application. Therefore, the study improves the quality information model for prefabricated nodes and the dragonfly algorithm by introducing principal component dimensionality reduction methods and multiple strategies through data modeling. Finally, a quality control model for anisotropic nodes and an optimization model for the load-bearing parameters of prefabricated wall panels are proposed. The experimental results showed that the control error of the nodal quality control model was as low as 0.9 mm. The displacement angle was as low as 0.037 rad. The maximum shear strength was 7.6 MPa. The minimum number of iterations of the parametric optimization model was 160 and the number of optimal solution strategies generated was 4500. The ratio of anti-overturning moment under wind and earthquake loads decreased by 0.45 and 0.13 compared with before optimization, respectively. Therefore, the proposed model can improve the load-bearing capacity and energy consumption capacity of nodes, and reduce control errors. The optimization of anisotropic nodes significantly enhances the seismic performance of shear walls. This offers a scientific foundation for designing and constructing prefabricated shear walls.

## 1. Introduction

In recent years, with the rapid development of the global construction industry, assembled buildings have gradually become the focus in the industry due to their high construction efficiency, controllable quality, as well as environmental protection and energy saving. However, the anisotropic nodes and bearing capacity of prefabricated wall panels in assembled shear wall structures are still technical bottlenecks restricting their widespread popularization [1]. For this reason, exploring the quality control of anisotropic nodes in shear wall structures and the bearing capacity of prefabricated wall panels has become an important task to ensure the safety and durability of building structures. At present, scholars at home and abroad have made significant progress in the research on anisotropic nodes and prefabricated wall panel forces. For example, finite element analysis and the most simplified control model are proposed, but there are still deficiencies in the complexity of data processing, the accuracy of model control, and the stability in practical applications. They fail to provide systematic optimization solutions. In order to solve the above problems, a quality control model for anisotropic nodes in prefabricated shear wall structures and an optimization model for bearing force parameters of prefabricated wall panels based on Principal Component Analysis (PCA) and Dragonfly Algorithm (DA) are proposed. The innovation of the study is that PCA and DA are combined for the first time. The efficiency and accuracy of the model is improved by multi-strategy dynamic adjustment algorithm, which fills the technological gap of the existing optimization methods in the optimization of high-dimensional data. The research aims to provide more scientifically optimization solutions for assembly building design and promote the technical progress in this field. This research is divided into four parts. The first part reviews existing research. The second part introduces how the anisotropic node quality control model and the prefabricated wall panel bearing force parameter optimization model are constructed, respectively. The third part tests the performances of the two types of models. The last part summarizes the article.

## 2. Literature review

In the research of prefabricated buildings, the anisotropic nodes of shear wall structures and the stress problems of prefabricated wall panels have always been key challenges that limit their widespread application. In this study, anisotropic nodes specifically refer to the connection nodes between shear wall members, which have significantly different responses in stiffness, shear capacity and deformation capacity due to forces in different directions, such as seismic and wind loads in the horizontal and vertical directions [2]. In order to improve the seismic performance and load carrying capacity of these structures, many scholars have carried out in-depth research. Optimization schemes are proposed for different nodes and wall panel connection forms. Firstly, for anisotropic nodes, S. D. Shen et al. aimed to achieve effective horizontal connections between the flange and web of prefabricated T-shaped shear walls and ensure the seismic performance of the nodes. A T-shaped shear wall panel anisotropic node control model was developed through finite element analysis after considering yield force and yield displacement. The anisotropic node strategy model enhanced the seismic demand of shear walls by adjusting its position and distribution [3]. M. Fan et al. verified the effect of different types of beam column joints on the static load of lightweight thermal insulation decorative wall panels. The failure modes, lateral stiffness, bearing ability, etc., were analyzed separately. They conducted internal force calculations on the steel frame. The L-shaped corner node significantly improved the node bearing ability, lateral stiffness, etc. This caused the plastic hinge of the node to move outward [4]. X. Wu et al. aimed to improve the effectiveness and reliability of shear connection nodes in prefabricated shear wall structural systems. A simplified constitutive model was proposed for a new type of steel shear connection node. The new node under this model had stable shear bearing capacity and superior energy dissipation capacity. The maximum error between its theory and simulation was only 7.67% [5]. To improve the accuracy and production efficiency of design in prefabricated shear wall structures, S. Li et al. introduced Building Information Modeling (BIM) and Tekla software to construct a design model of anisotropic nodes and three-dimensional steel bars. This model predicted the amount of steel reinforcement required for the production and construction of prefabricated components and the quantity of nodes. This promoted the intelligent development of prefabricated building design [6]. C. Mei et al. investigated the influence of anisotropic nodes and structural parameters on prefabricated double-sided connection composite shear walls’ seismic performance. A finite element model was established to test the seismic performance of shear walls under different anisotropic nodes. The composite column with T-shaped node, aspect ratio of 4, center distance of 150mm, and composite wall with thickness of 75mm had good seismic performance [7].

Secondly, regarding the bearing capacity of prefabricated wall panels, L. Meng et al. studied two types of vertical connections and conducted cyclic tests on four samples of cast-in-place concrete and vertical connections. Compared with ordinary concrete shear walls, the seismic performance index of self-compacting concrete pouring was significantly improved, with better experimental results [8]. Z. He et al. conducted experiments on directional beam column joints of reinforced prefabricated buildings. The grouted square hollow section sleeve connection axial static bearing capacity was analyzed. As the grouting length increased, the axial static bearing capacity of the specimen increased approximately linearly. When the grouting length was sufficient, the bearing capacity of the outer pipe cross-section was reached [9]. S. Shao et al. found that traditional shear walls typically exhibited smaller deformation capacity than frames under earthquakes. Therefore, an innovative prefabricated slotted composite shear wall was proposed. Compared with traditional shear walls, this new type of shear wall had the characteristics of concrete filled pipe wall sections, etc. The deformation, energy consumption, and construction efficiency of this new type of shear wall were significantly better than traditional shear walls [10]. G. Du et al. proposed a prefabricated steel active powder concrete component to enhance the load-bearing capacity of existing prefabricated wall panels. The axial displacement and other bearing capacity of this component under different conditions far exceeded those of traditional prefabricated panel components [11]. A. L. Zhang et al. proposed a method for separating core tube flange connection column nodes to guarantee prefabricated wall panel flange connections’ horizontal shear performance. At the same inter story displacement angle, the separated core tube connecting node had a fuller hysteresis curve and higher performance. High strength bolts had lower tensile strength [12].

In summary, significant progress has been made in optimizing the load-bearing capacity of anisotropic nodes and prefabricated wall panels in prefabricated shear wall structures at the current stage. The main technologies include finite element analysis, BIM, steel shear connection nodes, and reactive powder concrete components. These technologies show outstanding performance in improving structural performance, optimizing design, and enhancing seismic resistance. However, there are still issues such as precise control and optimization, complexity of data processing processes, and uncertainty in the construction process. Therefore, the study innovatively applies PCA and DA to the optimization design of prefabricated shear wall structures. By dynamically adjusting and optimizing through multiple strategies, more efficient and precise node control and parameter optimization are achieved.

## 3. Methods and materials

Quality control models for anisotropic nodes of prefabricated shear walls and optimization models for load-bearing parameters of prefabricated shear wall panels are constructed. Firstly, mathematical modeling is conducted on the quality control of anisotropic nodes. The Quality Information Model for Prefabricated Nodes (QIMPN) is introduced. Improvements are made based on PCA and DC. Secondly, a multi-objective model of bearing capacity parameters is constructed through mathematical modeling. DA is introduced with multiple strategies for improvement. Finally, a new type of optimized model for the load-bearing and stress parameters of prefabricated shear wall panels is proposed.

### 3.1. Quality control model for anisotropic nodes in prefabricated shear wall structures

**Fig 1:**
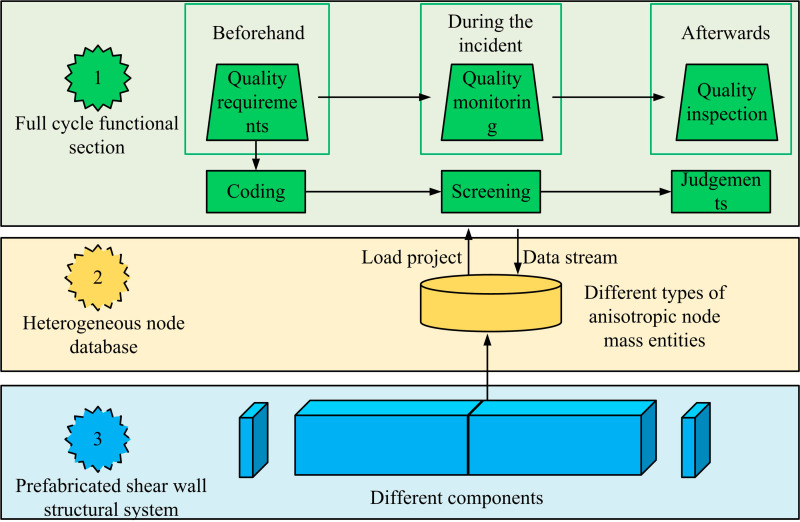
QIMPN framework diagram.

Anisotropic nodes refer to complex nodes formed in prefabricated shear wall structures due to different geometric shapes or connection methods of components such as walls, beams, and columns [13]. Meanwhile, due to the involvement of multiple materials and different component connections in anisotropic nodes, their construction and quality control have certain complexity and challenges [14]. Therefore, ensuring the quality of these nodes is directly related to the safety and durability of the entire building structure. In traditional construction, the quality control of anisotropic nodes mainly relies on experience and on-site inspection, but this method often has low accuracy and efficiency. To address these issues, the study introduces QIMPN. Fig 1 shows the model framework of QIMPN [15,16].

**Fig 2:**
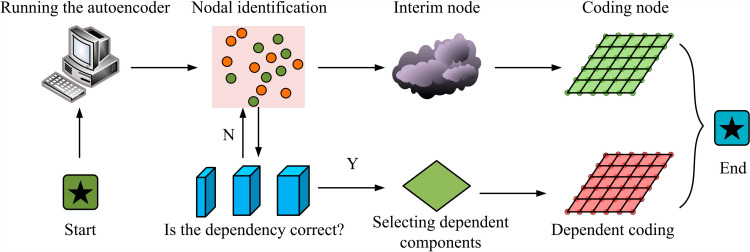
Node coding process.

In Fig 1, the entire QIMPN framework consists of three parts: full cycle function, anisotropic node database, and prefabricated shear wall structural system. The full cycle function is divided into three stages: pre-event, in event, and post-event, corresponding to three steps: node quality requirements, node quality monitoring, and node quality detection [17,18]. The prefabricated shear wall structure system is a building structure that transports prefabricated walls, floor slabs, and other components to the construction site for assembly after factory production. It includes prefabricated walls, prefabricated floors, prefabricated beams, prefabricated columns, prefabricated stairs and other components, as well as corresponding connection nodes, embedded accessories and connectors. QIMPN relies on its unique full cycle function to convert anisotropic nodes into entities and virtual entities, while combining with autoencoders to randomly encode nodes, achieving quality virtualization control [19,20]. Fig 2 shows the node encoding process.

In Fig 2, first, the auto-encoder is initialized and its initial parameters are adjusted. After completion, different types of nodes are identified and placed in an area. At this point, the label generator temporarily encodes different sets of nodes. By sequentially selecting the dependent components corresponding to the node, it is determined whether the node belongs to the corresponding component. If it is true, the dependent nodes are encoded and combined with the corresponding temporary nodes to form the final label node. If it is not true, the dependent components corresponding to the node are repeatedly selected. Although such anisotropic node quality control reduces the influence of some uncertainties, there is still a single quality control for coding and monitoring sessions, which can only rely on the existing node data for control. In view of this, the study introduces PCA to reduce the dimensionality of the entire process based on QIMPN. It is assumed that there are *m* quality indicators for anisotropic nodes, *n* samples for each indicator, and the quality matrix is X=m×n. To avoid changes in the importance of sample values caused by different dimensions of each indicator variable, the study first initializes the samples for dimensionless processing. The quality data matrix is simplified in the experiment, and this process is represented by equation (1).


$$\{\begin{array}{c} X=\left[\begin{array}{c} \begin{array}{ccc} x_{11} & \cdots & x_{1m} \end{array}\\ \begin{array}{ccc} \vdots & \ddots & \vdots \end{array}\\ \begin{array}{ccc} x_{n1} & \ldots & x_{nm} \end{array} \end{array}\right]\\ S_{i}=\sqrt{\frac{1}{n-1}\sum_{k=1}^{n}X_{nm}}\\ Y_{ij}=\frac{X_{nm}}{S_{i}}\\ Y=\left[\begin{array}{c} \begin{array}{ccc} Y_{11} & \cdots & Y_{1m} \end{array}\\ \begin{array}{ccc} \vdots & \ddots & \vdots \end{array}\\ \begin{array}{ccc} Y_{n1} & \ldots & Y_{nm} \end{array} \end{array}\right] \end{array}$$ (1)

In equation (1), both *i* and *j* are constants. *Y* represents the simplified quality matrix. $S_{i}$ means a quality matrix after dimensionless processing. In this process, by establishing covariance variance, the internal variance of quality indicators at each node is maximized, represented by equation (2).


$$C=\sum =\frac{1}{n-1}\sum_{t=1}^{n}y_{i}y_{i}^{T}=\frac{1}{n-1}Y^{T}Y$$ (2)

In equation (2), *T* refers to the data statistic. *n* refers to the sample size. *C* refers to the covariance matrix. After diagonalization, the solution of node quality indicators is transformed into solving the eigenvalues and eigenvectors of the matrix, represented by equation (3).


$$CP_{i}=\lambda_{i}p_{i}(i=1,2,3,m)$$ (3)

In equation (3), $P_{i}$ represents the feature vector. $\lambda_{i}$ represents the characteristic value. The key factors and indicators for node quality control in this constructed principal component model are represented by equation (4).


Z=FP+E (4)

In equation (4), *F* refers to the principal component score matrix. *E* is a residual matrix. *P* represents the principal component load matrix. After constructing the principal component matrix, a test statistic is established to assess its stability. The statistical quantity and its upper limit are represented by equation (5).


$$\{\begin{array}{c} T^{2}=Y\Lambda_{\lambda }P^{T}Y^{T}=\Lambda_{\lambda }\cdot t^{T}\\ T^{2}\max=\frac{k(n^{2}-1)}{n(n-k)}F_{\alpha }(k,n-k) \end{array}$$ (5)

**Fig 3:**
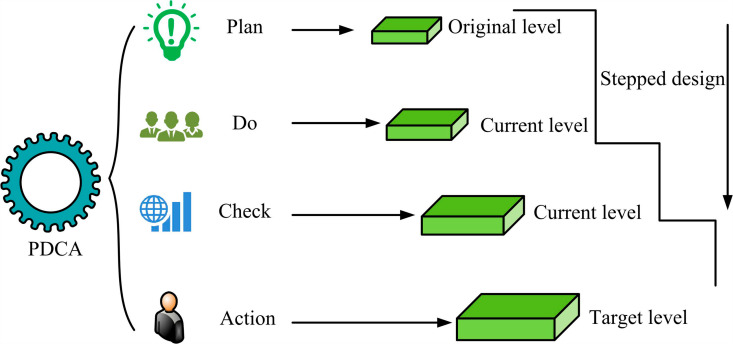
PCA-DC cycle improvement process.

In equation (5), $\Lambda_{\lambda }$ refers to a set of diagonal matrices. *α* represents the significance. *t* means the vector score in the principal component matrix. *k* represents the quantity of pivot elements. If too many quality control indicator elements are selected for nodes, it will lead to a decrease in the processing efficiency of QIMPN and PCA. The data noise inevitably affects the accuracy of node control. Therefore, the study introduces the Deming Cycle (DC) theory for optimization [21,22]. DC points out that quality improvement needs to be gradual, so that each quality control defect can be optimized by PCA-DC in Fig 3 [23].

**Fig 4:**
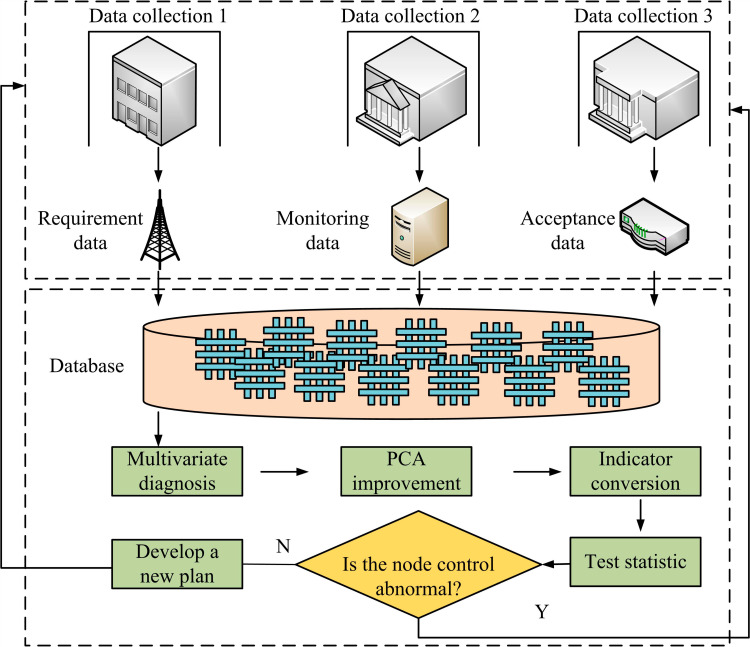
Quality control model for anisotropic nodes in prefabricated shear wall structures.

In Fig 3, PCA-DC undergoes bidirectional flow through four steps: planning, implementation, inspection, and processing. During the inspection phase, it is necessary to review the plan and implementation operations to check for any defects. During the processing phase, the optimal control scheme is further optimized step by step to meet higher control requirements. In summary, after combining QIMPN, PCA, and DC technologies, a quality control model for anisotropic nodes in prefabricated shear wall structures is finally proposed, as displayed in Fig 4.

In Fig 4, firstly, all anisotropic nodes of the prefabricated shear wall are scanned for three-dimensional positions and information by an external detector. After completion, QIMPN conducts quality requirements, node monitoring, and node acceptance for anisotropic nodes. After completion, a complete quality database is built. Then, the data of various anisotropic nodes in the database are subjected to multivariate data dimensionality reduction processing through PCA. Meanwhile, the indicators are transformed using matrix and principal component models and judged by test statistics. If there is an abnormality in the quality control of the opposite node, the improved method is identified and reverse transmitted to QIMPN for re-operation. If the quality control of anisotropic nodes is normal, a new quality improvement plan is further developed to gradually optimize the quality control effect.

### 3.2. Optimization of load-bearing parameters for prefabricated wall panels based on improved dragonfly algorithm

**Fig 5:**
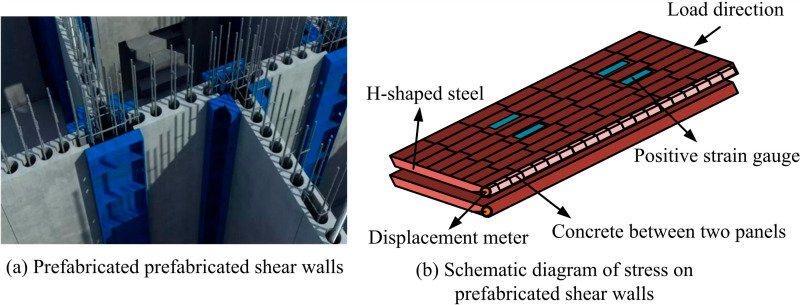
Prefabricated shear wall panels and their load-bearing schematic diagram.

Assembled precast shear wall panels are structural elements that are pre-fabricated in a factory and assembled at the construction site, which are widely used in high-rise buildings and seismic design [24,25]. Prefabricated shear wall panels have excellent seismic performance and can effectively resist horizontal forces such as earthquakes and wind loads [26]. The assembled prefabricated shear wall panel and its load-bearing schematic are shown in Fig 5.

Fig 5(a) shows the on-site installation diagram of prefabricated shear wall panels. Fig 5(b) is a schematic diagram of the stress on prefabricated shear wall panels. Prefabricated shear wall panels are affected by various factors during the load-bearing process. These factors include the strength of wall panel materials, steel reinforcement configuration, design of connection nodes, load type and distribution, wall panel size and thickness, etc. [27,28]. A mathematical model for the stress of prefabricated shear wall panels is constructed using mathematical modeling. The rationality of stress includes torsion cycle ratio, torsion displacement ratio, structural shear weight ratio, axial compression ratio, inter story stiffness ratio, and inter story displacement angle. To facilitate subsequent unified operations, the study adopts a dimensionless approach to handle these factors. The constrained torsion cycle ratio and torsion displacement ratio are represented by equation (6).


$$\{\begin{array}{c} m_{1}=T_{L}\leq RI\cdot T_{1}\\ m_{2}=\frac{\Delta u_{\max}}{\Delta u}\leq RI \end{array}$$ (6)

In equation (6), RI refers to the randomness indicator. $T_{L}$ and $T_{1}$ correspond to the first natural vibration period of the structure for translational and automatic motion, respectively. Δu is the average displacement of building floors. $\Delta u_{\max}$ represents the maximum displacement. When $m_{1}=T_{L}\leq RI\cdot T_{1}$, it is assumed that the self-oscillation period $T_{L}$ of the shear wall cannot exceed the product of the reference period $T_{1}$ and the stochasticity index RI to ensure that the seismic performance of the structure meets the standard. When $m_{2}=\frac{\Delta u_{\max}}{\Delta u}\leq RI$, it is used to limit the ratio of the maximum displacement to the average displacement, thus controlling the deformation capacity of the wall. The constraint equations for the shear to weight ratio and axial compression ratio of the structure are shown in equation (7).


$$\{\begin{array}{c} m_{3}=V_{Eki}>\lambda \sum_{j=1}^{n}G_{j}\\ m_{4}=\frac{N}{Af_{c}}\leq \left|U\right| \end{array}$$ (7)

In equation (7), *λ* refers to the shear coefficient. $G_{j}$ refers to the gravitational load of the *j* th floor. $V_{Eki}$ refers to the horizontal floor shear force of the *i* th floor. $f_{c}$ refers to the axial compressive strength of the structure. *A* refers to the cross-sectional area of the column. *N* refers to the axial force design value of the column structure. $\left|U\right|$ represents the limit value of the axial compression ratio. $m_{3}=V_{Eki}>\lambda \sum_{j=1}^{n}G_{j}$ represents the shear stress model of a shear wall under multiple loads. $m_{4}=\frac{N}{Af_{c}}\leq \left|U\right|$ is used to describe the stress distribution during axial force. The axial force of the shear wall is limited by the bearing capacity per unit area $f_{c}$ and the load distribution *N*. The constraints of inter story stiffness ratio and inter story displacement angle are represented by equation (8).


$$\{\begin{array}{c} m_{5}=\frac{K_{i+1}}{K_{i}}\leq \left|\tau \right|,\frac{K_{i}}{K_{i-1}}\leq \left|\tau \right|\\ m_{6}=\Delta u_{c}\leq \left|\theta \right|h \end{array}$$ (8)

In equation (8), $\left|\tau \right|$ represents the boundary value of the displacement stiffness ratio. $K_{i+1}$ and $K_{i-1}$ correspond to the upper and lower displacement stiffness of the *i* th floor, respectively. $\left|\theta \right|$ represents the boundary value of inter story displacement angle. *h* refers to the height of the floor. $\Delta u_{c}$ represents the maximum elastic inter story displacement of the floor. $m_{5}=\frac{K_{i+1}}{K_{i}}\leq \left|\tau \right|,\frac{K_{i}}{K_{i-1}}\leq \left|\tau \right|$ is used to describe the inter story stiffness ratio of wall panels under shear deformation, assuming that the change rate of stiffness is limited by the allowable shear stress $\left|\tau \right|$. $m_{6}=\Delta u_{c}\leq \left|\theta \right|h$ is used to control the relationship between the inter story displacement angle $\left|\theta \right|$ and the floor height *h* to ensure that the displacement angle is within the design specification range. However, these formulations are based on static analysis and simplified mechanical models, which may exhibit limitations for complex nonlinear, and random seismic loading. Factors that contribute to the rationality of stress in the above structure are summarized. At this point, the multi-objective function of the force on the prefabricated shear wall panel is represented by equation (9).


$$M_{c}{\text{=\{m}}_{\text{1}}{\text{,m}}_{\text{2}}{\text{,m}}_{\text{3}}{\text{,m}}_{\text{4}}{\text{,m}}_{\text{5}}{\text{,m}}_{\text{6}}{\text{,m}}_{\text{7}}{\text{,m}}_{\text{8}}\text{\}}$$ (9)

**Fig 6:**
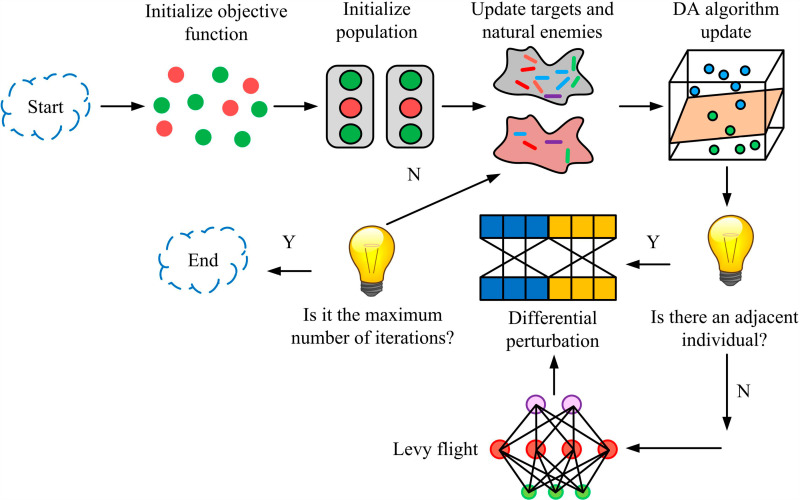
IDA flowchart.

This study introduces DA to better seek the optimal solution for the multi-objective function. DA is a novel biomimetic optimization method that simulates the flight path and behavior of dragonflies to achieve separation, pairing, aggregation, and avoidance behaviors. Compared with other algorithms, DA is simpler and faster with fewer parameter settings. However, in long-term optimization operations, if the target source positions are all in the same local solution, the individual dragonfly is prone to falling into the local optimal solution [29]. Therefore, the study continues to introduce multiple strategies for dynamic adjustment. Fig 6 shows the optimized DA process.

In Fig 6, first, the objective function and parameters are initialized and set. After completion, the population position is initialized using the Elite Opposition-Based Learning (EOBL) strategy. Secondly, the physical target and natural enemy positions are updated, namely the optimal and worst solution positions. Then, the inertia factor and adaptive inertia weight values of DA are updated. The search range radius is updated. If there are adjacent individuals, position updates and random differential perturbations are performed. If there are no adjacent individuals, Levy flight is used for position update and random score perturbation is performed simultaneously. Finally, if the perturbation result reaches the maximum number of iterations, the output ends. If the maximum number of iterations is not reached, additional iterations are added. The physical target and natural enemy positions are updated repeatedly. During this process, the calculation of EOBL is represented by equation (10).


$$x_{t}^{'}=rand(x_{min},x_{max})-(x_{i}-x_{min})$$ (10)

In equation (10), $x_{i}^{'}$ refers to the position of the reverse individual. $x_{i}$ represents the current individual’s position. $x_{\max}$ and $x_{\min}$ correspond to the upper and lower limits of the search space, respectively. $x_{\min}$ represents a random number within an interval. The adaptive inertia weight is represented by equation (11).


$$\omega =\omega_{\max}-\frac{\omega_{\max}-\omega_{\min}}{\eta_{\max}}\eta_{i}$$ (11)

In equation (11), $\eta_{i}$ refers to the *i* th iteration. $\eta_{i}$ represents the maximum number of iterations. $\omega_{\max}$ and $\omega_{\min}$ correspond to the maximum and minimum inertia weights, respectively. To balance the global search capability and local development capability of the algorithm, the inertia factor is updated, represented by equation (12).


$$\omega '=\omega_{\max}\times \cos\frac{\pi \times \eta_{i}}{2\eta_{\max}}+\omega_{\min}\times (1-\cos\frac{\pi \times \eta_{i}}{2\eta_{\max}})$$ (12)

In equation (12), when $\omega_{\min}$ and $\omega_{\max}$ are 0.4 and 0.9, respectively, the inertia factor can reach the minimum and maximum values. The position update of random differential perturbation is represented by equation (13).


$$x_{i}(t+1)=x_{i}(t)+F\cdot (x_{r}1(t)-x_{r}2(t))$$ (13)

**Fig 7:**
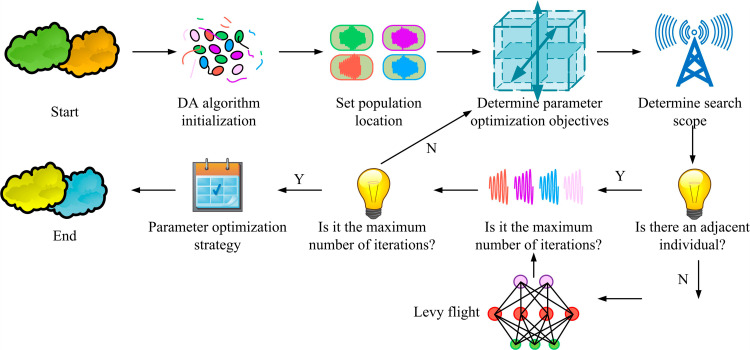
Optimization model for stress parameters of matched shear wall panels.

In equation (13), $x_{i}(t+1)$ refers to the new position of the *i* th individual in the (t+1)th generation. *F* refers to the scaling factor. $x_{i}(t+1)$ and $x_{r}2(t)$ correspond to the positions of two randomly selected individuals in the GG generation. In summary, a final optimization model for the stress parameters of prefabricated shear wall panels is constructed, as displayed in Fig 7.

In Fig 7, first, the objective function and parameter values of DA are initialized. Next, the population position is initialized. Then, the factors and indicators of the stress on the prefabricated shear wall panel are determined. The target search radius is updated. If there are adjacent individuals, the position information of the best individual is updated and its fitness function is calculated. If there are no adjacent individuals, the individual position is updated through Levy flight. Finally, if the maximum iteration is reached at this point, the optimal force parameter control strategy for prefabricated shear wall panels is output. If the iteration does not reach the maximum value, additional iterations will be added to redefine the stress coefficients and indicators of the prefabricated shear wall panels within the new range. Subsequent calculations will continue.

The experimental design is based on an assembly residential building project in Chenghua District, Chengdu City as the test background, and the test includes the performance verification of the quality control model of anisotropic nodes and the optimization model of prefabricated wall panels bearing force parameters. The tests firstly conducted ablation experiments on I- and L-shaped nodes to evaluate the quality control error, inter-story displacement angle and shear strength as the core indexes respectively, and to compare the performance optimization effects of the three models of QIMPN, QIMPN-PCA and QIMPN-PCA-DC. Subsequently, in the test of the optimization model of prefabricated wall panels with bearing force parameters, the multi-objective function optimization strategy is adopted to verify the stability and bearing capacity of IDA algorithm under wind and seismic loads, and the core indexes include the number of iterations, the number of optimal solution strategies, the tilting moment resistance, and the structural quality coefficient, etc., and the performance improvement before and after the optimization is finally compared.

## 4. Results

This study is tested in a suitable experimental environment to validate the newly proposed model. Firstly, the experiment is conducted through ablation testing and indicator comparison. The quality control model for anisotropic nodes in prefabricated shear wall structures is tested through simulation verification. Then, the optimization model for the load-bearing capacity parameters of prefabricated wall panels is validated based on algorithm parameter optimization strategies, function index testing, and simulation testing.

### 4.1. Testing of quality control model for anisotropic nodes in prefabricated shear wall structures

A suitable experimental environment is established to verify the quality control model for the newly proposed anisotropic nodes in prefabricated shear wall structures. The CPU is set to Intel Core i7, the GPU is NVIDIA GeForce, the memory is 32GB, the development environment is Python, and the Windows operating system is used. A prefabricated residential building project in Chenghua District, Chengdu City is taken as the testing background. The total building height of the project is 36.7m, with a building area of 5443.9m^2^, a single story of 457.3m^2^, a floor height of 3.7m, 5 inner wall panels, and 37 laminated panels. It is rated as an AA level prefabricated building. AA type wall nodes are used to test the objects. Anisotropic nodes are further divided into I-shaped, I-shaped, L-shaped, T-shaped, and cross-shaped. Table 1 shows the detailed classification of I-shaped and L-shaped nodes.

**Table 1:** I/L type node quality entity type.

Heterosexual node type	Provisional code	Node quality description
I-shaped/L-shaped	AA-Q8-1	Vertical reinforcement connections for edge construction
	AA-Q8-2	All connections of vertically distributed steel bars in shear walls
	AA-Q8-3	Partial connection of vertically distributed reinforcement in shear walls
	AA-Q8-4	Shear wall shear connection structure
	AA-Q8-5	Shear wall variable section reinforcement distribution structure
	AA-Q8-6	Shear wall vertical reinforcement top construction
	AA-Q8-7	Horizontal post-cast construction
	AA-Q8-8	Post-cast ring beam construction
	AA-Q8-9	Horizontal backing strip reinforcement construction
	AA-Q8-10	Reinforcement construction of post-cast ring beam
	AA-Q8-11	Horizontal backing strip with longitudinal beam reinforcement overlap
	AA-Q8-12	Longitudinal reinforcement overlap in backing ring

**Fig 8:**
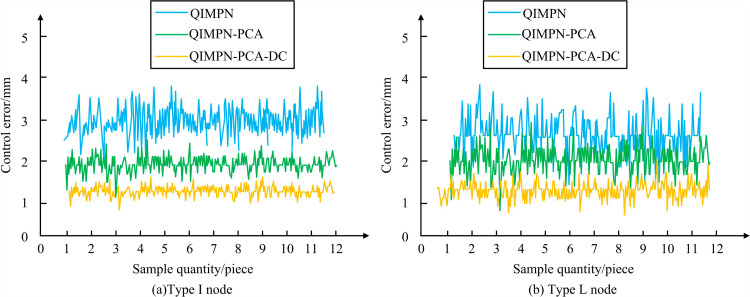
Ablation test results of QIMPN-PCA-DC model.

After constructing the I-shaped node quality entity classification standard, considering quantitative and qualitative indicators for testing, non-quantitative indicators are temporarily excluded. The study conducts ablation testing on the proposed QIMPN-PCA-DC prefabricated shear wall structure quality control model for anisotropic nodes. The quality control error is tested, as shown in Fig 8.

**Fig 9:**
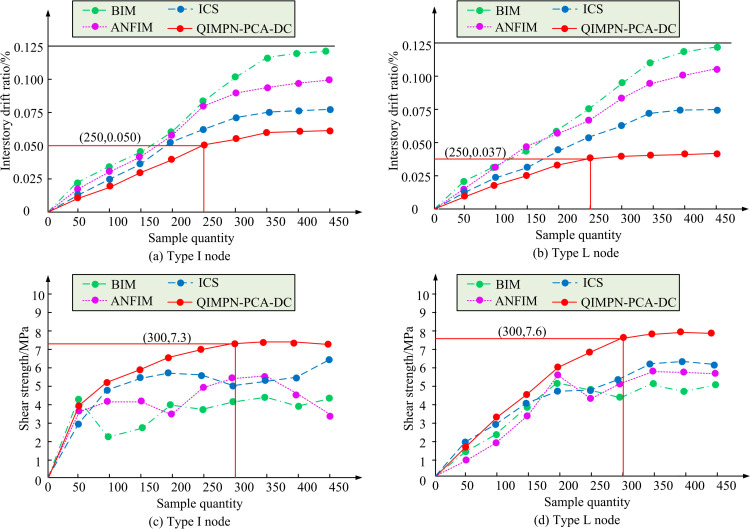
Test results of inter story displacement angle and shear strength of different models.

Fig 8(a) shows the control error test results of each module for QIMPN-PCA-DC in the I-shaped node quality classification system. Fig 8(b) shows the control error test results of each module for QIMPN-PCA-DC in the L-shaped node quality classification system. In the quality control test of the I-shaped node, the control error of the individual QIMPN module fluctuated between 2.5-3.5mm. The control error after introducing PCA fluctuated within 1.5-2.5mm. The control error after incorporating DC was within 1-1.5mm. In addition, in the quality control of L-shaped nodes, the control error of the individual QIMPN module fluctuated within 2.5-3.8mm. The control range of QIMPN-PCA was within 1-2.8mm. The QIMPN-PCA-DC module had the best control error performance, with a control range of 0.9-1.7mm. Each module in the proposed anisotropic node quality control model had a promoting effect, which optimized the overall performance. In addition, the study introduces popular quality control models for prefabricated shear wall nodes of the same type, such as BIM, Adaptive Neuro-Fuzzy Inference Modle (ANFIM), and Intelligent Construction System (ICS). Inter story displacement angle and shear strength are displayed in Fig 9.

**Fig 10:**
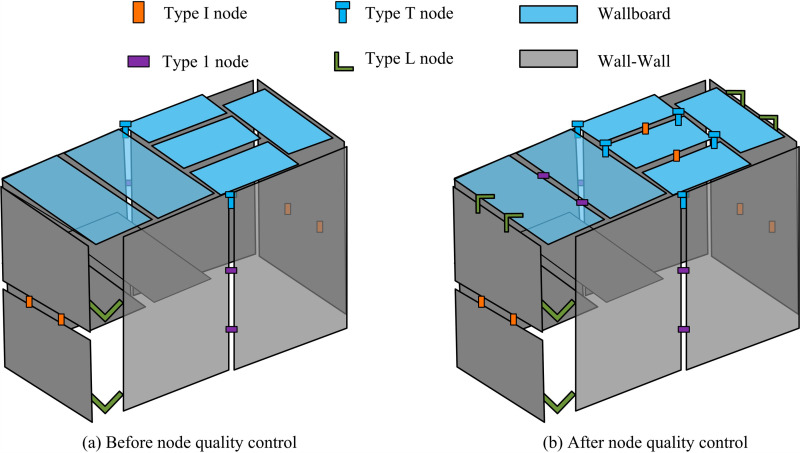
Comparison of quality control optimization for anisotropic nodes before and after optimization.

Fig 9(a) shows the inter story displacement angle test results of four models at I-shaped nodes. Fig 9(b) shows the inter story displacement angle test results of four models at L-shaped nodes. Fig 9(c) shows the shear strength test results of four models at I-shaped nodes. Fig 9(d) shows the shear strength test results of four models at L-shaped nodes. A smaller inter story displacement angle means that the deformation of the structure under horizontal loads is smaller, which helps to improve the safety and stability of the structure and reduce the risk of structural damage. The test results of the proposed model were generally low, with minimum inter story displacement angles of 0.05 and 0.037 in quality control of I-shaped and L-shaped nodes, respectively. Generally speaking, the higher the shear strength, the better, because higher shear strength can improve the bearing capacity and seismic performance of shear walls, enhance the safety and durability of structures. The shear strength of BIM, ICS, and ANFIM models fluctuated significantly with the test samples. Under the node quality control of the proposed model, the shear strength gradually increased, with the highest values in I-shaped and L-shaped nodes being 7.3MPa and 7.6MPa, respectively. In actual projects, the shear strength of ordinary concrete was usually between 2 and 5 MPa, depending on the concrete mix ratio and construction technology. The shear strength of high-strength concrete reached more than 6 MPa. Therefore, the 7.6MPa shear strength achieved by the research model far exceeds the performance of traditional concrete, indicating that the optimization method proposed in this paper can significantly improve the shear bearing capacity of the structure. Finally, the study uses a single story house of a prefabricated residential building project in Chenghua District, Chengdu City as a benchmark to draw the quality control results, as displayed in Fig 10.

Fig 10(a) shows the strength expression results of the housing project before optimizing the quality control of anisotropic nodes. Fig 10(b) shows the expression of the strength of the housing project after optimizing the quality control of anisotropic nodes. From this comparison figure, the BIM technology of the foundation before optimization is able to achieve the wall-wall anisotropic node control of the foundation though. However, in assembled shear walls, this foundation BIM focuses more on information integration and static management, and its responsiveness and monitoring accuracy are relatively weak for node control that requires dynamic adjustment. On the contrary, the optimized quality control model for anisotropic nodes achieves multi-node control through the autoencoder of QIMPN. Meanwhile, these complex wall and panel nodes are unified using PCA-DC. This enhances the stability of shear wall anisotropic node connections throughout the entire building project.

### 4.2. Optimization model testing of load-bearing and stress parameters for prefabricated wall panels

**Fig 11:**
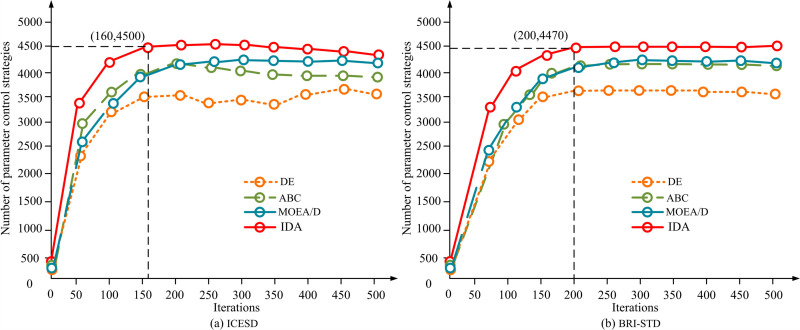
The performance of four algorithms on different datasets.

The proposed IDA prefabricated wall panel bearing and stress parameter optimization model is validated. This study uses the International Civil Engineering Simulation Database (ICESD) and the Building Research Institute Structural Test Dataset (BRI-STD) as test data sources in the same software environment. ICESD contains more than 20,000 data, covering the force and performance parameters of key structural components such as shear walls, including wind load, seismic load, axial compression ratio, etc. BRI-STD contains a variety of structural performance data collected from actual building tests, totaling about 15,000 data, which is mainly used to validate the seismic performance and load carrying capacity of the structure. The K-nearest neighbor algorithm is used to fill the corresponding missing data with samples that are most similar to the missing values, while normalizing the data before model training. In the experiments, the DA and IDA optimization models are used to carry out the force analysis of the shear wall structure, and the optimal parameter values are selected. The validity and accuracy of the proposed models are verified by comparing with simulation data and physical experiment data. The research first tests the algorithm part, namely IDA. Differential Evolution (DE), Artificial Bee Colony (ABC), and Multi-Objective Evolutionary Algorithm based on Decomposition (MOEA/D), which are popular algorithms of the same type, are introduced. Fig 11 shows the test results.

**Fig 12:**
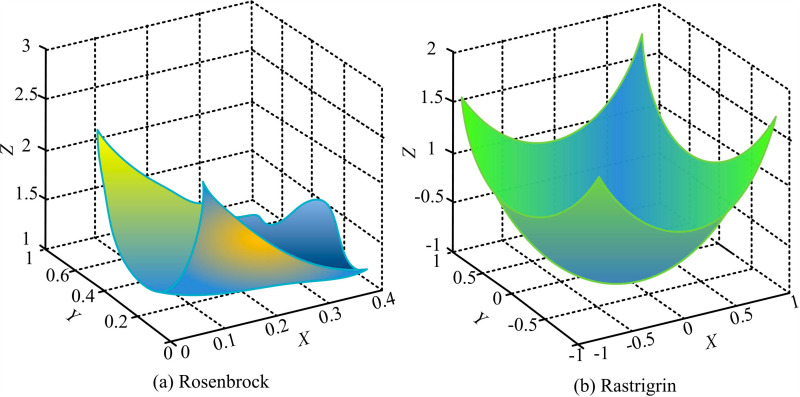
Testing function diagram of QIMPN-PCA-DC.

Fig 11(a) shows the operational results of four algorithms in multi-objective problems on the ICESD dataset. Fig 11(b) shows the operational results of four algorithms in the BRI-STD multi-objective problem. The proposed IDA had a faster search speed in the early stage. The reason may be that IDA has high sensitivity in the early stages through multi-strategy optimization. The minimum number of iterations for IDA was 160. The optimal solutions were 4500. In BRI-STD, IDA still performed the best, with a minimum of 200 iterations and nearly 4470 optimal solutions. In summary, the proposed IDA had better computational power and fewer iterations compared with the other three types of algorithms. In addition, the study tests IDA using two different testing functions, including the Rosenbrock unimodal sphere function and the Rastigrin multimodal function. The dimension of the above test functions is set to 100, as displayed in Fig 12.

**Fig 13:**
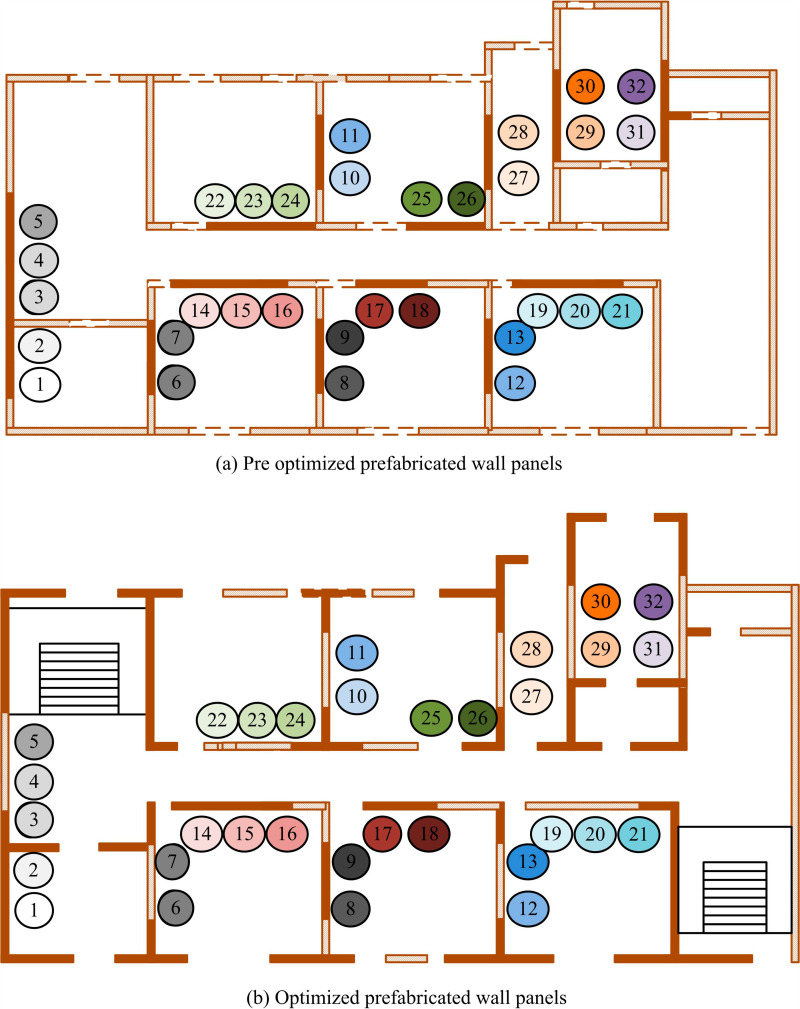
Comparison of configuration effects before and after optimizing wall making parameters.

Fig 12(a) shows the test results of IDA on the Rosenbrock unimodal sphere function. Fig 12(b) shows the test results of IDA on the Rastigrin multimodal sphere function. After testing IDA, the optimization range of the Rosenbrock function was within [0.13, 0.3], which was the yellowish part. After testing IDA, the optimization range of the Rastigrin function was within [-0.53, 0.53], which was the blue part. Compared with basic DA, the proposed IDA had higher accuracy, stronger stability, and certain feasibility. In addition, the study also uses a single story house of a prefabricated residential building project in Chenghua District, Chengdu as a benchmark to compare the optimized building models to better highlight the advantages of the research model. The design includes 32 design variables. The design variables numbered 2, 3, 16, 21, 24, 30, and 32 represent wall elements. Other numbered design variables represent virtual wall elements. Fig 13 shows the wall configuration before and after optimization.

Fig 13(a) shows the prefabricated wall configuration before parameter optimization. Fig 13(b) shows the prefabricated wall configuration after parameter optimization. In the multi-objective structural design of prefabricated shear walls with optimized parameters, there was a significant increase in non-load-bearing walls and a decrease in load-bearing walls, effectively reducing construction costs. The types of components also decreased compared with the wall configuration before optimization, resulting in a significant improvement in construction costs and efficiency. To further test the performance of the proposed prefabricated wall panel optimization model in terms of bearing capacity parameters, dynamic tests are carried out by simulating the effects of seismic loading. The stability, self-oscillation period, structural mass coefficients and inter storey displacement angles of the optimized structure are analyzed. Table 2 shows the test results.

**Table 2:** Multiple indicator test results.

Indicator	Style	DA simulation result	IDA simulation result	Experimental result
Structural stability (under wind load)	Wind load	21.98	21.53	21.61
Structural stability (under earthquake load)	Earthquake load	6.08	6.21	6.19
Structural mass Factor (X direction)	X mass factor (%)	95.71	95.92	95.84
Structural mass Factor (Y direction)	Y mass factor (%)	94.56	95.46	95.3
Natural period (first translational period)	First translational cycle	1.2083	0.8367	0.8423
Natural period (first rotational period)	First rotational cycle	1.2467	0.8436	0.849

In Table 2, in the simulation results of structural stability under wind load, the DA was 21.98 and the IDA was 21.53, which were very close to the experimental result of 21.61, indicating that the simulation model was able to accurately predict the structural performance under wind load. Under seismic loading, the difference between the IDA simulation result of 6.21 and the experimental result of 6.19 was only 0.02, which further indicated that the model had high accuracy in predicting the load carrying capacity. In addition, the comparison of the self-resonance period and mass factor of the structure also showed a good agreement between the simulation and the experimental results, especially in the self-resonance period. The IDA values of 0.8367 and 0.8436 were within a reasonable error range compared with the experimental results of 0.8423 and 0.8490. This indicated that the IDA-optimized model was able to predict the dynamic response of shear walls more accurately in real engineering.

## 5. Discussion

In recent years, assembled shear wall structures have been widely used in the field of construction, especially in high-rise buildings, where the advantages of seismic performance and construction efficiency have attracted much attention. The study proposes an anisotropic node quality control model and a prefabricated wall panel load bearing force parameter optimization model by combining PCA and DA. In the experiments, the optimized model achieved shear strengths of 7.3 MPa and 7.6 MPa at the I- and L-shaped nodes, respectively, which far exceeded the 2-5 MPa interval for ordinary concrete wall panels, showing excellent shear resistance. In addition, the minimum displacement angles were 0.05 rad and 0.037 rad, respectively, indicating that the optimized model significantly reduced the horizontal deformation of the structure under seismic loading and improved the stability of the structure. Compared with traditional methods such as BIM and ANFIM, the QIMPN-PCA-DA shows better performance in all key indicators. In terms of material properties, the optimized model ensures the deformation control of wall panels and nodes under loads by increasing the nodal stiffness and shear strength. The high-strength concrete material involved in the study not only enhances the strength of the wall panels, but also effectively alleviates the stress concentration problem by optimizing the ductility of the nodes, which further improves the seismic performance of the structure. Compared with the research of T. Luo et al. whose proposed anisotropic node model for shear wall panels mainly focuses on the optimization of yield force and yield displacement, the study achieves significant progress in shear strength and deformation control through the comprehensive optimization of material properties and node parameters [30].

In summary, the optimization model performs excellently in enhancing the shear capacity and seismic performance of shear wall structures, and its practical application value is demonstrated by experimental and simulation results. However, the limitation of the study is that construction errors and long-time performance changes of materials have not been fully considered. Therefore, future research can further combine field experiments to explore the effects of construction errors, material variability and other factors, and extend the applicability of the model under complex loading conditions.

## 6. Conclusion

There are issues with the load-bearing capacity of shear wall structures and prefabricated wall panels in prefabricated buildings. Therefore, the study adopted a mathematical modeling approach. A quality control model for anisotropic nodes and an optimization model for the load-bearing parameters of prefabricated wall panels were constructed by combining PCA and IDA, respectively. In the quality control testing of I-shaped and L-shaped nodes, the control error of QIMPN-PCA-DA fluctuated between 1.5-2.5mm and 0.9-1.7mm, respectively. The minimum inter story displacement angles were 0.05 and 0.037, respectively. The highest values of shear strength were 7.3MPa and 7.6MPa, respectively. Compared with before optimizing node quality control, multi-node control was achieved through QIMPN’s autoencoder under optimized model control. In addition, in the ICESD dataset, IDA had a minimum of 160 iterations and 4500 optimal solutions. In the BRI-STD dataset, the minimum number of iterations for IDA was 200, with nearly 4470 optimal solutions. Compared with the other three types of algorithms, IDA had better computational power and fewer iterations. After testing the Rosenbrock unimodal sphere function and Rastigrin multimodal function, compared with the basic DA, the proposed IDA had a smaller difference in optimal solutions and better convergence accuracy. Meanwhile, the ratio of anti-overturning moment of IDA under wind and earthquake loads decreased by 0.45 and 0.13, respectively. In summary, these two types of models proposed significantly improve the overall structural performance of prefabricated shear walls. However, the research mainly focuses on the technical and theoretical aspects of optimization, without fully considering real-world constraints such as manufacturing tolerances, structural errors, and material variability. In addition, variations in material properties, such as inconsistencies in the quality of concrete or steel reinforcement, can lead to limitations in the theoretical results of the model in practice. Future research can continue to explore the application effects of the above models in practical prefabricated building projects. Models can be iteratively optimized for specific challenges encountered during on-site construction.

## Supporting information

S1 File Minimal data set definition. (DOCX)
