# Supplementary material for: Optimization of load-bearing parameters for anisotropic nodes and prefabricated wall panels in prefabricated shear wall structures
Source: PLoS One. 2025 Mar 17;20(3):e0318521. doi: 10.1371/journal.pone.0318521 (PMC11913284; doi:10.1371/journal.pone.0318521)
Supplement: S1 File — (DOCX) [file pone.0318521.s001.docx]

**Figure 8 Ablation test results of QIMPN-PCA-DC model**

| Sample Quantity | QIMPN (Type I node error/mm) | QIMPN-PCA (Type I node error/mm) | QIMPN-PCA-DC (Type I node error/mm) | QIMPN (Type L node error/mm) | QIMPN-PCA (Type L node error/mm) | QIMPN-PCA-DC (Type L node error/mm) |
| --- | --- | --- | --- | --- | --- | --- |
| 1 | 3.1 | 2.0 | 1.3 | 3.4 | 2.3 | 1.1 |
| 2 | 3.2 | 2.3 | 1.4 | 3.5 | 2.5 | 1.2 |
| 3 | 3.4 | 2.4 | 1.5 | 3.6 | 2.4 | 1.3 |
| 4 | 3.1 | 2.1 | 1.2 | 3.2 | 2.2 | 1.1 |
| 5 | 3.3 | 2.5 | 1.5 | 3.4 | 2.6 | 1.5 |
| 6 | 3.0 | 2.3 | 1.3 | 3.3 | 2.4 | 1.3 |
| 7 | 3.5 | 2.5 | 1.5 | 3.7 | 2.8 | 1.7 |
| 8 | 3.3 | 2.4 | 1.4 | 3.5 | 2.5 | 1.2 |
| 9 | 3.4 | 2.3 | 1.4 | 3.8 | 2.6 | 1.4 |
| 10 | 3.2 | 2.1 | 1.3 | 3.4 | 2.5 | 1.3 |
| 11 | 3.1 | 2.2 | 1.2 | 3.6 | 2.4 | 1.1 |
| 12 | 3.4 | 2.4 | 1.5 | 3.7 | 2.7 | 1.2 |

**Figure 9 Test results of inter story displacement angle and shear strength of different models**

| Sample quantity | 50 | 100 | 150 | 200 | 250 | 300 | 350 | 400 | 450 |
| --- | --- | --- | --- | --- | --- | --- | --- | --- | --- |
| BIM (Type I node - Drift angle %) | 0.125 | 0.175 | 0.28 | 0.225 | 0.25 | 0.275 | 0.34 | 0.32 | 0.33 |
| ANFIM (Type I node - Drift angle %) | 0.12 | 0.16 | 0.19 | 0.21 | 0.24 | 0.26 | 0.28 | 0.32 | 0.31 |
| ICS (Type I node - Drift angle %) | 0.13 | 0.165 | 0.195 | 0.22 | 0.245 | 0.27 | 0.29 | 0.31 | 0.32 |
| QIMPN-PCA-DC (Type I node - Drift angle %) | 0.05 | 0.075 | 0.19 | 0.125 | 0.15 | 0.175 | 0.24 | 0.225 | 0.25 |
| BIM (Type L node - Drift angle %) | 0.125 | 0.15 | 0.175 | 0.2 | 0.225 | 0.25 | 0.275 | 0.3 | 0.32 |
| ANFIM (Type L node - Drift angle %) | 0.12 | 0.145 | 0.17 | 0.195 | 0.22 | 0.245 | 0.265 | 0.29 | 0.31 |
| ICS (Type L node - Drift angle %) | 0.13 | 0.155 | 0.18 | 0.205 | 0.23 | 0.255 | 0.275 | 0.295 | 0.315 |
| QIMPN-PCA-DC (Type L node - Drift angle %) | 0.037 | 0.062 | 0.087 | 0.112 | 0.137 | 0.162 | 0.187 | 0.212 | 0.237 |
| BIM (Type I node - Shear strength / MPa) | 2.1 | 2.5 | 3.2 | 4.4 | 4.5 | 5.6 | 6.2 | 6.5 | 7.3 |
| ANFIM (Type I node - Shear strength / MPa) | 2.2 | 2.7 | 3.2 | 4.2 | 4.7 | 5.2 | 6.2 | 6.7 | 7.2 |
| ICS (Type I node - Shear strength / MPa) | 2.1 | 2.6 | 3.1 | 4.1 | 4.6 | 5.1 | 6.1 | 6.6 | 7.1 |
| QIMPN-PCA-DC (Type I node - Shear strength / MPa) | 3.3 | 3.5 | 4.2 | 4.5 | 5.5 | 6.6 | 6.5 | 7.4 | 7.3 |
| BIM (Type L node - Shear strength / MPa) | 2.4 | 2.6 | 3.1 | 4.8 | 4.4 | 5.3 | 5.8 | 6.4 | 7.8 |
| ANFIM (Type L node - Shear strength / MPa) | 2.3 | 2.8 | 3.3 | 4.3 | 4.8 | 5.3 | 6.3 | 6.8 | 7.3 |
| ICS (Type L node - Shear strength / MPa) | 2.2 | 2.7 | 3.2 | 4.2 | 4.7 | 5.2 | 6.2 | 6.7 | 7.2 |
| QIMPN-PCA-DC (Type L node - Shear strength / MPa) | 3.1 | 3.6 | 4.1 | 4.6 | 5.6 | 6.1 | 6.6 | 7.1 | 7.6 |

**Figure 11 The performance of four algorithms on different datasets**

| Iterations | DE (ICESD) | ABC (ICESD) | MOEA/D (ICESD) | IDA (ICESD) | DE (BRI-STD) | ABC (BRI-STD) | MOEA/D (BRI-STD) | IDA (BRI-STD) |
| --- | --- | --- | --- | --- | --- | --- | --- | --- |
| 50 | 3000 | 3200 | 3300 | 4000 | 2800 | 2900 | 3100 | 3800 |
| 100 | 3500 | 3600 | 3700 | 4200 | 3300 | 3400 | 3500 | 4000 |
| 150 | 3700 | 3800 | 3900 | 4400 | 3600 | 3700 | 3800 | 4200 |
| 200 | 3800 | 3900 | 4000 | 4500 | 3700 | 3800 | 3900 | 4400 |
| 250 | 3900 | 4000 | 4100 | 4500 | 3800 | 3900 | 4000 | 4470 |
| 300 | 4000 | 4100 | 4200 | 4500 | 3900 | 4000 | 4100 | 4470 |
| 350 | 4100 | 4200 | 4300 | 4500 | 4000 | 4100 | 4200 | 4470 |
| 400 | 4200 | 4300 | 4400 | 4500 | 4100 | 4200 | 4300 | 4470 |
| 450 | 4300 | 4400 | 4500 | 4500 | 4200 | 4300 | 4400 | 4470 |
| 500 | 4400 | 4500 | 4600 | 4500 | 4300 | 4400 | 4470 | 4470 |

**Figure 12 Testing Function Diagram of QIMPN-PCA-DC**

| X (Rosenbrock) | Z (Rosenbrock) | X (Rastrigrin) | Z (Rastrigrin) |
| --- | --- | --- | --- |
| 0.13 | 1.0 | -0.5 | -1.5 |
| 0.14 | 1.1 | -0.5 | -1.4 |
| 0.15 | 1.2 | -0.4 | -1.2 |
| 0.18 | 1.4 | -0.3 | -1.2 |
| 0.22 | 1.6 | -0.3 | -0.8 |
| 0.22 | 1.8 | -0.2 | -0.6 |
| 0.25 | 2.1 | 0.0 | -0.4 |
| 0.27 | 2.3 | 0.2 | -0.2 |
| 0.33 | 2.5 | 0.5 | 0.0 |
